# Combining methylated SDC2 test in stool DNA, fecal immunochemical test, and tumor markers improves early detection of colorectal neoplasms

**DOI:** 10.3389/fonc.2023.1166796

**Published:** 2023-08-09

**Authors:** Tao Zeng, Zhongchao Huang, Xufa Yu, Li Zheng, Tao Liu, Boyu Tian, Siyu Xiao, Jiahui Huang

**Affiliations:** ^1^ Department of Clinical Laboratory, The Sixth Affiliated Hospital, Sun Yat-sen University, Guangzhou, Guangdong, China; ^2^ Biomedical Innovation Center, The Sixth Affiliated Hospital, Sun Yat-sen University, Guangzhou, Guangdong, China; ^3^ Department of Gastroenterology, The Sixth Affiliated Hospital, Sun Yat-sen University, Guangzhou, Guangdong, China; ^4^ Department of Clinical Laboratory, Collaborative Innovation Center for Cancer Medicine, Sun Yat-sen University Cancer Center, Guangzhou, Guangdong, China; ^5^ State Key Laboratory of Oncology in South China, Collaborative Innovation Center for Cancer Medicine, Sun Yat-sen University Cancer Center, Guangzhou, Guangdong, China

**Keywords:** SDC2, fecal immunochemical test, colorectal neoplasms, tumor markers, combined testing

## Abstract

**Objective:**

To explore the value of testing methylated *SDC2* (SDC2) in stool DNA combined with fecal immunochemical test (FIT) and serum tumor markers (TM) for the early detection of colorectal neoplasms.

**Methods:**

A total of 533 patients, including 150 with CRC (67 with early-stage CRC), 23 with APL, 85 with non-advanced adenomas and general polyps, and 275 with benign lesions and healthy controls. SDC2 was detected by methylation-specific PCR, FIT (hemoglobin, Hb and transferrin, TF) was detected by immunoassay, and the relationships between SDC2, FIT, and clinicopathological features were analyzed. Pathological biopsy or colonoscopy were used as gold standards for diagnosis, and the diagnostic efficacy of SDC2 combined with FIT and TM in CRC and APL evaluated using receiver operating characteristic (ROC) curves.

**Results:**

SDC2 positive rates in early-stage CRC and APL were 77.6% (38/49) and 41.2% (7/17), respectively, and combination of SDC2 with FIT increased the positive rates to 98.0% (48/49) and 82.4% (14/17). The positive rates of SDC2 combined with FIT assay in the APL and CRC groups at stages 0-IV were 82.4% (14/17), 85.7% (6/7), 100% (16/16), 100% (26/26), 97.4% (38/39), and 100% (22/22), respectively. Compared to the controls, both the CRC and APL groups showed significantly higher positive detection rates of fecal SDC2 and FIT (χ2 = 114.116, P < 0.0001 and χ2 = 85.409, P < 0.0001, respectively). Our results demonstrate a significant difference in the qualitative methods of SDC2 and FIT for the detection of colorectal neoplasms (McNemar test, P < 0.0001). ROC curve analysis revealed that the sensitivities of SDC2 and FIT, alone or in combination, for the detection of early CRC and APL were 69.9%, 86.3%, and 93.9%, respectively (all P<0.0001). When combined with CEA, the sensitivity increased to 97.3% (P<0.0001).

**Conclusions:**

SDC2 facilitates colorectal neoplasms screening, and when combined with FIT, it enhances detection. Furthermore, the combination of SDC2 with FIT and CEA maximizes overall colorectal neoplasm detection.

## 1 Introduction

Colorectal cancer (CRC) is a common malignant tumor of the digestive system and one of the most frequently occurring malignant tumors in China (1). Abnormal cells carrying mutations or methylation signals are shed into the stool and can be detected, making stool a theoretically better specimen than blood for early detection of CRC (2–4). Advanced adenoma (AA) and advanced serrated polyp (ASP) are currently considered important advanced precancerous lesions (APL) of CRC, because they increase the risk of CRC disease and death (4–9). Progression from APL to CRC is thought to occur over 5–10 years, which provides a valuable window of time for disease diagnosis and intervention (10). Current guidelines recommend CRC screening methods, such as colonoscopy and fecal occult blood test (FOBT), but colonoscopy requires specialized physicians and is somewhat invasive, making it unsuitable for large-scale population screening. Therefore, there is an urgent need for non-invasive tests to improve screening rates. The fecal immunochemical test (FIT) is non-invasive, simple, highly sensitive and is now widely used in the clinic. In recent years, fecal DNA testing has developed rapidly, and fecal DNA methylation testing has undergone numerous prospective clinical trials in China, confirming its suitability for CRC screening (11–13).Some studies have shown that the specificity of fecal SDC2 methylation (SDC2) was very high, or even > 90%, and had good diagnostic agreement with colonoscopy results, indicating that SDC2 is a suitable marker for CRC screening (11, 14–16); however, the clinical application of SDC2 remains somewhat controversial (17, 18). There have been few studies analyzing the relationships between clinicopathological features and SDC2 results. Moreover, comparisons of SDC2 testing with early screening for FIT and combined screening are infrequent. In this study, we analyzed the usefulness of fecal SDC2 in colorectal neoplasm screening by testing it in patients who underwent colonoscopy, and we combined this with clinical and pathological data. We also compared the results of SDC2 testing with the routine clinical application of FIT to investigate whether it could detect more early-stage CRC and APL. It is well-established that combining multiple markers for screening can improve tumor detection rates. For example, blood protein markers combined with DNA mutations can be used to detect various early-stage cancers (19). Considering the widespread use of serum tumor markers (TM) in clinical practice, we also investigated the efficacy of combining SDC2 and FIT with TM testing for early-stage CRC and APL detection.

## 2 Methods

### 2.1 Patients and methods

This was a retrospective case-control study. Outpatients and inpatients who underwent colonoscopy and/or pathological examination at The Sixth Affiliated Hospital of Sun Yat-sen University, Guangzhou, China, from March 2019 to September 2022 were recruited to this study. A total of 533 patients were included: 150 cases of CRC, comprising 138 with stage I–IV and 12 cases of carcinoma *in situ* and intramucosal CRC; 23 cases of APL, including 21 cases of AA and 2 cases of ASP; 85 with non-AA and general polyps (mainly inflammatory polyps and hyperplastic polyps); and 275 with benign lesions and healthy controls. Among patients with CRC, 67 had early-stage disease, including 12 with stage 0, 22 with stage I, and 33 with stage II; 79 had advanced CRC (stage III and IV); and 4 cases had disease of unknown stage.

All patients were first-time outpatients or inpatients who had not received relevant treatment (including drugs and surgery), and colonoscopy or pathological findings were used as the gold standards for diagnosis, with CRC diagnosed according to the Chinese Colorectal Cancer Diagnosis and Treatment Standard (20) and CRC TNM staging according to the TNM staging system for colorectal cancer in the 8th edition of the AJCC (21).

Study exclusion criteria were as follows: 1. suffering from other systemic malignancies; 2. previous history of colorectal neoplasms, or had undergone colonoscopy resection or surgical treatment of CRC; 3. had undergone comprehensive treatment for CRC; 4. no pathological or colonoscopy findings, including cases in which polyps were found but not treated; 5. patients with incomplete clinical information and unknown diagnosis, of which eight cases with neither SDC2 nor FIT findings were also excluded; 5. cases with inadequate bowel preparation (inadequate cases); or 6. gastrointestinal mesenchymal tumors and presacral tumors.

This study was approved by the Ethics Committee of The Sixth Affiliated Hospital of Sun Yat-sen University (Ethics No. 2022ZSLYEC-508).

### 2.2 Instruments and methods

The instructions for specific tests are provided in the attached Instructions Summary; a brief description of each test method is also provided below.

Fecal SDC2 was detected using the human SDC2 gene methylation detection kit (methylation-specific PCR (22) method) from Creative Biosciences (Guangzhou) Co., Ltd. First, samples were extracted using magnetic beads and then treated with sulfite; following which, the methylated SDC2 gene would not be transformed, while methylated SDC2 could be amplified by specific primers, with ACTB as the internal reference gene. Samples were judged to be positive when the Ct value of the ACTB gene was ≤ 36 and that for SDC2 was ≤ 38. The specific detection principles and steps, as well as influencing factors, were detailed in a previous report (14).

FIT is an immunoassay used to analyze fecal occult blood, including tests for hemoglobin (Hb) and transferrin (TF), using reagents and instruments from Keyu Biosciences (Zhuhai) Co., Ltd. The presence of two red lines on the test card (i.e., a quality control line and detection line) indicated a positive occult blood test. The sensitivity values of the Hb and TF tests were ≥ 100 ng/ml and ≥ 40 ng/ml, respectively, and the Hb and TF tests did not cross-react; positivity for either test was defined as a positive FIT result. Serum TM (included CEA, CA125, CA19-9, CA15-3, and AFP) were tested using Abbott Alinity and corresponding reagents and instruments, CEA results exceeding 5ng/ml are considered positive.

The kits used have obtained Chinese registration certificates and manufacturing licenses, and all operations are carried out in strict compliance with the operating manual. In addition, the specimens undergo indoor quality control before being tested.

### 2.3 Statistical analysis

IBM SPSS 26.0 statistical software was used for data analysis. Count data are expressed as frequencies and percentages, and the χ2 test was used for comparison between two groups. McNemar’s test was utilized to analyze the consistency and differences between the two qualitative diagnostic test methods. The sensitivity and specificity for qualitative results are calculated using a crosstab, while the sensitivity and specificity for quantitative results are calculated using logistic regression. The diagnostic efficacy of each diagnostic technique for CRC and APL was evaluated using receiver operating characteristic (ROC) curves. Mann-Whitney tests or t-tests were used for comparisons of quantitative data. All tests were two-sided. P < 0.05 was considered statistically significant.

## 3 Results

### 3.1 Patient characteristics

A total of 533 cases were included in the study: 173 cases of CRC and APL in the disease group, including 150 cases of CRC (67 cases of early-stage CRC) and 23 cases of APL; and 360 disease controls and healthy controls, including 85 cases of non-AA and general polyps and 275 cases of benign lesions and healthy controls.

### 3.2 Stool DNA test of methylated SDC2

The positive rate of fecal SDC2 in CRC was 79.3%, including 77.6% in early-stage CRC, 82.0% in advanced CRC, and 41.2% in APL, with a gradual increasing trend of positive rate with disease severity: APL < early-stage CRC < advanced CRC (**Table 1**).

**Table 1:** Clinical data from cases and controls undergoing fecal SDC2 and FIT testing.

		SDC2		FIT^1^ (Hb and TF)		SDC2 and FIT^4^	
	No.	Positive NO.	Sensitivity (95% CI) %	Positive NO.	Sensitivity (95% CI) %	Positive NO.	Sensitivity (95% CI) %
Colorectal cancer	111	88	79.3 (70.5 - 86.4)	104	93.7 (87.4 - 97.4)	108	97.3 (92.3 - 99.4)
Early-Stage CRC	49	38	77.6 (63.4 - 88.2)	46	93.8 (83.1 - 98.7)	48	98.0 (89.1 - 99.9)
Stage 0	7	6	85.7 (42.1 - 99.6)	4	57.1 (18.4 - 90.1)	6	85.7 (42.1 - 99.6)
Stage I	16	15	93.8 (69.8 - 99.8)	16	100 (79.4 - 100.0)	16	100 (79.4 - 100.0)
Stage II	26	17	65.4 (44.3 - 82.8)	26	100 (86.8 - 100.0)	26	100 (86.8 - 100.0)
Advanced CRC	61	50	82.0 (70.0 - 90.6)	58	95.1 (86.3 - 99.0)	60	98.4 (91.2 - 100.0)
Stage III	39	30	76.9 (60.7 - 88.9)	37	94.9 (82.7 - 99.4)	38	97.4 (86.5 - 99.9)
Stage IV	22	20	90.9 (70.8 - 98.9)	21	95.5 (77.2 - 99.9)	22	100 (84.6 - 100.0)
Stage unknown	1	0	0	0	0	0	0
APL^2^	17	7	41.2 (18.4 - 67.1)	11	64.7 (38.3 - 85.8)	14	82.4 (56.6 - 96.2)
Early-stage CRC and APL	66	45	68.2 (55.6 - 79.1)	57	86.4 (75.7 - 93.6)	62	93.9 (85.2 - 98.3)
CRC and APL	128	95	74.2 (65.7 - 81.5)	115	89.8 (83.3 - 94.5)	122	95.3 (90.1 - 98.3)
			Specificity (95% CI)		Specificity (95% CI)		Specificity (95% CI)
Non-advanced adenoma and general polyp	24	4	83.3 (62.6 - 95.3)	6	75.0 (53.3 - 90.2)	9	62.5 (40.6 - 81.2)
Benign lesions and negative results on colonoscopy^3^	156	21	86.5 (80.2 - 91.5)	61	60.9 (52.8 - 68.6)	74	52.6 (44.4 - 60.6)

^1^Any single positive result (Hb or TF) was considered a positive result. ^2^Including advanced adenoma and ≥ 1 cm serrated polyps. ^3^Benign lesions, including colitis, ulcerative colitis, Crohn’s disease, intestinal tuberculosis, diverticulum, mixed hemorrhoids, anal fistula, etc. ^4^If either SDC2 or FIT has a positive result, it is considered positive. CI, Confidence interval.

### 3.3 Consistency of fecal SDC2 with colonoscopy or pathological findings

A total of 518 cases were included in comparison of the results of SDC2 testing with those of colonoscopy or pathology findings, including 158 cases of CRC and APL. The Kappa value was 0.54 (χ2 = 153.422, P < 0.001), indicating that SDC2 was consistent with the results of colonoscopy with moderate concordance.

### 3.4 Comparison of fecal SDC2 with FIT

The positive rates of fecal SDC2 in the CRC plus APL group and the early-stage CRC plus APL group were 74.2% and 68.2%, respectively, while those for routine FIT were 89.8% and 86.4%, respectively (**Table 1**).

Conventional FIT demonstrated a higher positive detection rate for early-stage and advanced CRC and APL than fecal SDC2. In comparison to 180 healthy and disease controls, both the CRC and APL groups had significantly higher positive detection rates of fecal SDC2 and FIT (χ2 = 114.116, P < 0.0001 and χ2 = 85.409, P < 0.0001, respectively). Additionally, significant differences were observed in the positive detection rates of the early-stage CRC and APL groups (χ2 = 69.640, P < 0.0001 and χ2 = 46.462, P < 0.0001, respectively).

In comparison to the differences in positivity rates between different groups, we were curious about the consistency and differences in positivity rates between SDC2 and FIT qualitative methods in CRC and APL (methodological comparison). Using the McNemar test, our analysis revealed notable distinctions in positivity rates between the two methods (Difference: 20.13%, 95% CI: 13.91 - 26.35, P < 0.0001). We also found differences in early-stage CRC and APL groups (Difference: 21.95%, 95% CI: 14.64 - 29.26, P < 0.0001). Our results demonstrate a significant difference in the qualitative methods of SDC2 and FIT for the detection of colorectal neoplasms. Furthermore, the combination of these two methods enhances the positive detection rate.

### 3.5 Combined fecal SDC2 and FIT

The combined detection rates of fecal SDC2 and FIT were 95.3% (122/128) in CRC plus APL, and 93.9% (62/66) in early-stage CRC plus APL (**Table 1**). Compared with the individual detection methods, the combined test significantly increased the positive detection rates of early-stage CRC, advanced CRC, and APL (98.0%, 98.4%, and 82.4%, respectively), suggesting that the two methods are complementary, and can detect more patients with APL and CRC when used in combination.

### 3.6 Associations between fecal SDC2 and FIT results and clinicopathological findings

#### 3.6.1 CRC histological type: adenocarcinoma versus mucinous adenocarcinoma

**Figure 1:**
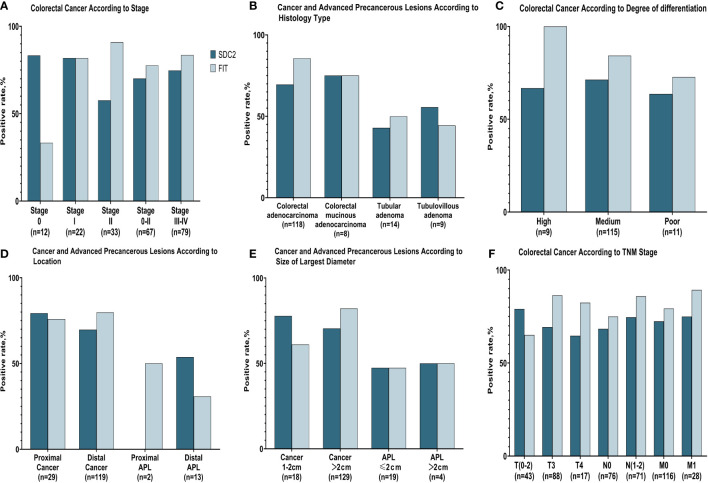
Positive rates of fecal SDC2 and FIT for detecting colorectal cancer and advanced precancerous lesions. **(A)** Positive rates of SDC2 and FIT for detecting different stages of CRC. **(B)** Positive rates of SDC2 and FIT for detecting CRC and APL with different histology types. **(C)** Positive rates of SDC2 and FIT for detecting CRC with different levels of differentiation. **(D)** Positive rates of SDC2 and FIT for detecting CRC and APL at different lesion sites. **(E)** Positive rates of SDC2 and FIT for detecting CRC and APL lesions of differing maximum diameters. **(F)** Positive rates of SDC2 and FIT for detecting CRC with different T, N, and M stages.

Adenocarcinoma accounted for the majority of CRC cases included in this study, with only a small proportion being mucinous adenocarcinoma. Both SDC2 and FIT yielded higher positivity rates in adenocarcinoma than in mucinous adenocarcinoma, but their positivity rates did not differ significantly between the two types. Additionally, the combined test improved the detection rate for the two types (**Table 2**; **Figure 1**).

**Table 2:** Comparisons of fecal SDC2 and FIT positive rates with different pathological results in patients with CRC.

		SDC2	FIT (Hb and TF)	SDC2 and FIT^4^		
	No.	Positive rate	Positive rate	Positive rate	χ2	P value
Histological type^1^					5.583	0.140
Adenocarcinoma	90	77.8% (70/90)	97.8% (88/90)	98.9% (89/90)		
Mucinous adenocarcinoma	7	71.4% (5/7)	85.7% (6/7)	85.7% (6/7)		
Degree of differentiation^2^					4.355	0.168
Highly and Medium	94	79.8% (75/94)	97.9% (92/94)	98.9% (93/94)		
Poor	9	66.7% (6/9)	88.9% (8/9)	88.9% (8/9)		
Location^3^					0.771	1.000
Proximal	22	86.4% (19/22)	95.5% (21/22)	100% (22/22)		
Distal	88	77.3% (68/88)	93.2% (82/88)	96.6% (85/88)		
TNM Stage						
T					2.807	0.156
0 - 2	28	89.3% (25/28)	85.7% (24/28)	92.9% (26/28)		
3 - 4	83	75.9% (63/83)	96.4% (80/83)	98.8% (82/83)		
N					0.343	1.000
0	55	76.4% (42/55)	92.7% (51/55)	96.4% (53/55)		
1 - 2	55	81.8% (45/55)	96.4% (53/55)	98.2% (54/55)		
M					0.509	1.000
0	88	77.3% (68/88)	94.3% (83/88)	97.7% (86/88)		
1	22	90.9% (20/22)	95.5% (21/22)	100% (22/22)		

^1^Histological type: adenocarcinoma, mucinous adenocarcinoma, adenocarcinoma combined with mucinous adenocarcinoma, micropapillary carcinoma, indolent cell carcinoma, squamous carcinoma, etc. ^2^Highly differentiated included highly differentiated and highly-moderately differentiated. Moderately differentiated included moderately differentiated and moderately-poorly differentiated. Poorly differentiated included poorly differentiated, mucinous adenocarcinoma. ^3^Proximal included cecum, ascending colon, hepatic flexure, transverse colon. Distal included splenic flexure, descending colon, sigmoid colon, rectum. ^4^If either SDC2 or FIT has a positive result, it is considered positive. The chi-square test was utilized to compare differences between two groups in a qualitative test. When the expected frequency in the chi-square test is less than 5, a modified chi-square test, also known as Fisher’s exact test, was used.

#### 3.6.2 Degree of CRC differentiation: highly and medium versus poor

The positive detection rate for fecal SDC2 or conventional FIT was low in poorly differentiated CRC, with 66.7% and 88.9%, respectively. Conversely, in medium and highly differentiated CRC, the positive detection rate for fecal SDC2 or FIT was higher, with 79.8% and 97.9%, respectively. There was no significant difference in the positivity rate of the combined SDC2 and FIT assay between medium and highly differentiated CRC compared to poorly differentiated CRC (χ2 = 4.355, P =0.168).

#### 3.6.3 Proximal versus distal CRC

Both fecal SDC2 and FIT had higher positive rates in proximal tumors than in distal tumors. There was no significant difference in the positivity rate of the combined SDC2 and FIT assay between proximal CRC compared to distal CRC (χ2 = 0.771, P =1.000). The combined fecal SDC2 and FIT test increased the positive detection rate for both proximal and distal tumors, resulting in a 100% positive rate for proximal tumors and a 96.6% positive rate for distal tumors.

#### 3.6.4 TNM stage

The T stage is mainly related to the depth of tumor infiltration. The positive rate of SDC2 gradually decreased as the depth of infiltration increased, from 89.3% at T0-2 to 75.9% at T3-4. Conversely, the positivity rate for FIT was higher at T3-4 (96.4%) than at T0-2 (85.7%). There was no significant difference in the positivity rate of the combined SDC2 and FIT assay between T0-2 stage compared to T3-4 stage (χ2 = 2.807, P =0.156). The combined test increases the positive detection rate for both T0-2 and T3-4 staging. The positive rates of SDC2 and FIT were higher in CRC with lymph node metastasis (N1–2) than in CRC without metastasis, suggesting that the detection rates of both SDC2 and FIT increase when lymph node metastasis is present. The combined test improved the detection rate of N stage, with the positive detection rate for CRC with lymph node metastasis reaching 98.2%. SDC2 and FIT showed higher positive rates in CRC with metastases than in those without. Specifically, when the two were tested in combination, the positivity rate for FIT was 97.7% for M0 and 100% for M1.

### 3.7 Diagnosis efficacy of fecal SDC2, FIT, and serum CEA, alone and in combination

**Figure 2:**
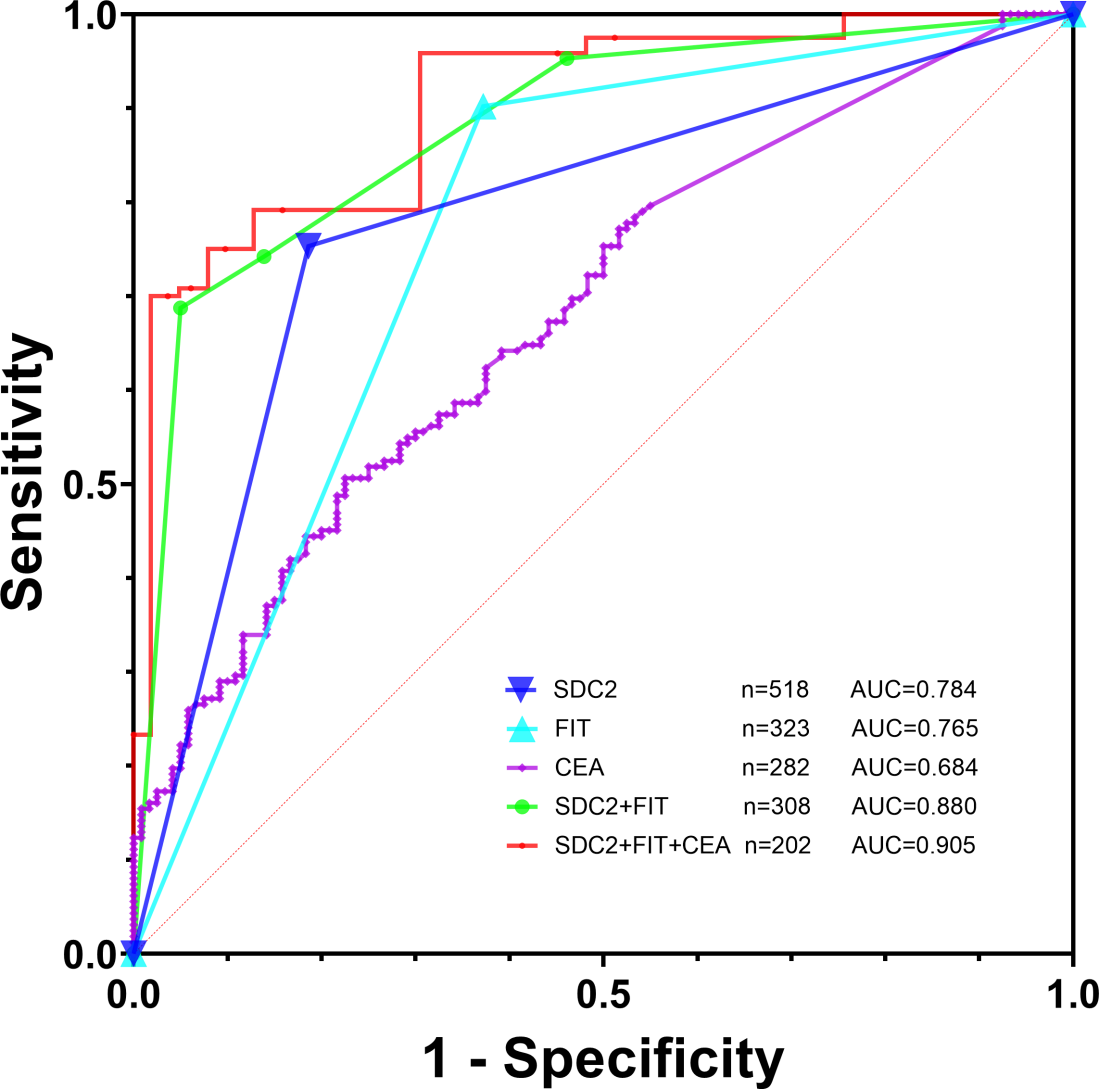
Efficacy of fecal SDC2, FIT, and tumor markers for CRC and APL diagnosis.

**Figure 3:**
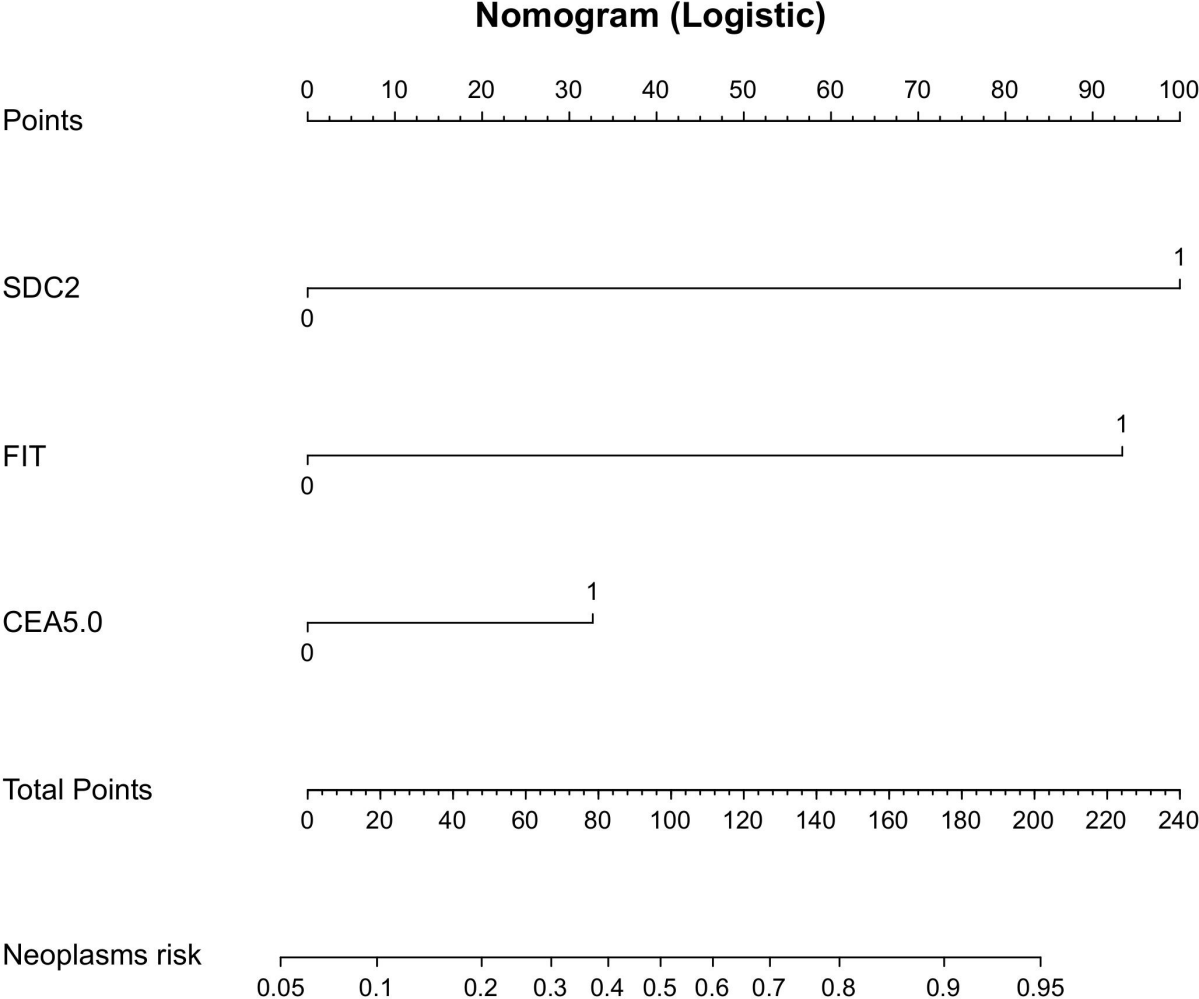
Nomogram-based prediction model for the risk of developing CRC and APL using logistic regression.

1. To investigate the diagnosis efficacy of relevant indicators, samples were categorized into a disease group consisting of CRC plus APL cases and a control group consisting of non-AA and general polyps, benign lesions, and healthy controls. The analysis of SDC2’s diagnosis efficacy for CRC plus APL revealed a sensitivity of 75.3%, specificity of 81.4%, and AUC of 0.784 (**Table 3**; **Figure 2**). Conversely, the sensitivity, specificity, and AUC values for FIT in detecting CRC plus APL were 90.2%, 62.8%, and 0.765, respectively. These data demonstrate that SDC2 exhibits lower sensitivity, but higher specificity, than FIT. Combination of SDC2 with conventional FIT resulted in detection efficacy of 68.8 sensitivity, 95.0% specificity, and AUC 0.880. Further, the detection efficacy of SDC2 combined with conventional FIT and serum CEA generated sensitivity, specificity, and AUC values of 70.0%, 96.3%, and 0.905, respectively. The joint test initially employs logistic regression to build the prediction curve, followed by ROC curve analysis to estimate the area under the curve. The coefficients for the independent variables, SDC2 and FIT, are 2.81602 and 2.63004, respectively. The coefficient for the CEA dependent variable is 0.92039. Finally, the model’s coefficient for the constant term is -2.7346. Based on the above list of coefficients, logistic regression can be calculated with the equation: p = e^ (β0 + β1 * x1 + β2 * x2)/(1 + e^ (β0 + β1 * x1 + β2 * x2)), where β0, β1, and β2 correspond to the constant term, SDC2 and FIT coefficients, respectively. We have created nomographs based on logistic regression, which are visually presented for the reference of clinicians (**Figure 3**).

**Table 3:** Efficacy of fecal SDC2, FIT, and serum CEA for CRC and APL diagnosis.

	No.	Sensitivity (%)	Specificity (%)	AUC (95% CI)	P value
SDC2	518	75.3	81.4	0.784 (0.746–0.818)	< 0.0001
FIT	323	90.2	62.8	0.765 (0.715–0.810)	< 0.0001
CEA	282	50.6	77.5	0.684 (0.627–0.738)	< 0.0001
SDC2 + FIT^1^	308	68.8	95.0	0.880 (0.839–0.914)	< 0.0001
SDC2 + FIT + CEA^2^	202	70.0	96.3	0.905 (0.856–0.941)	< 0.0001
SDC2 + FIT^3^	308	95.3	53.9	0.746 (0.694–0.794)	< 0.0001
SDC2 + FIT + CEA^3^	202	97.5	48.8	0.731 (0.665–0.791)	< 0.0001

FIT included fecal Hb and TF. The sensitivity and specificity of detecting quantitative CEA alone depend on logistic regression. ^1^Use logistic regression to build prediction curves and ROC curve analysis to calculate the area under the curve. ^2^CEA results exceeding 5ng/ml are considered positive, use logistic regression to build prediction curves and ROC curve analysis to calculate the area under the curve. ^3^Result was considered positive if any one of them has a positive result.

**Figure 4:**
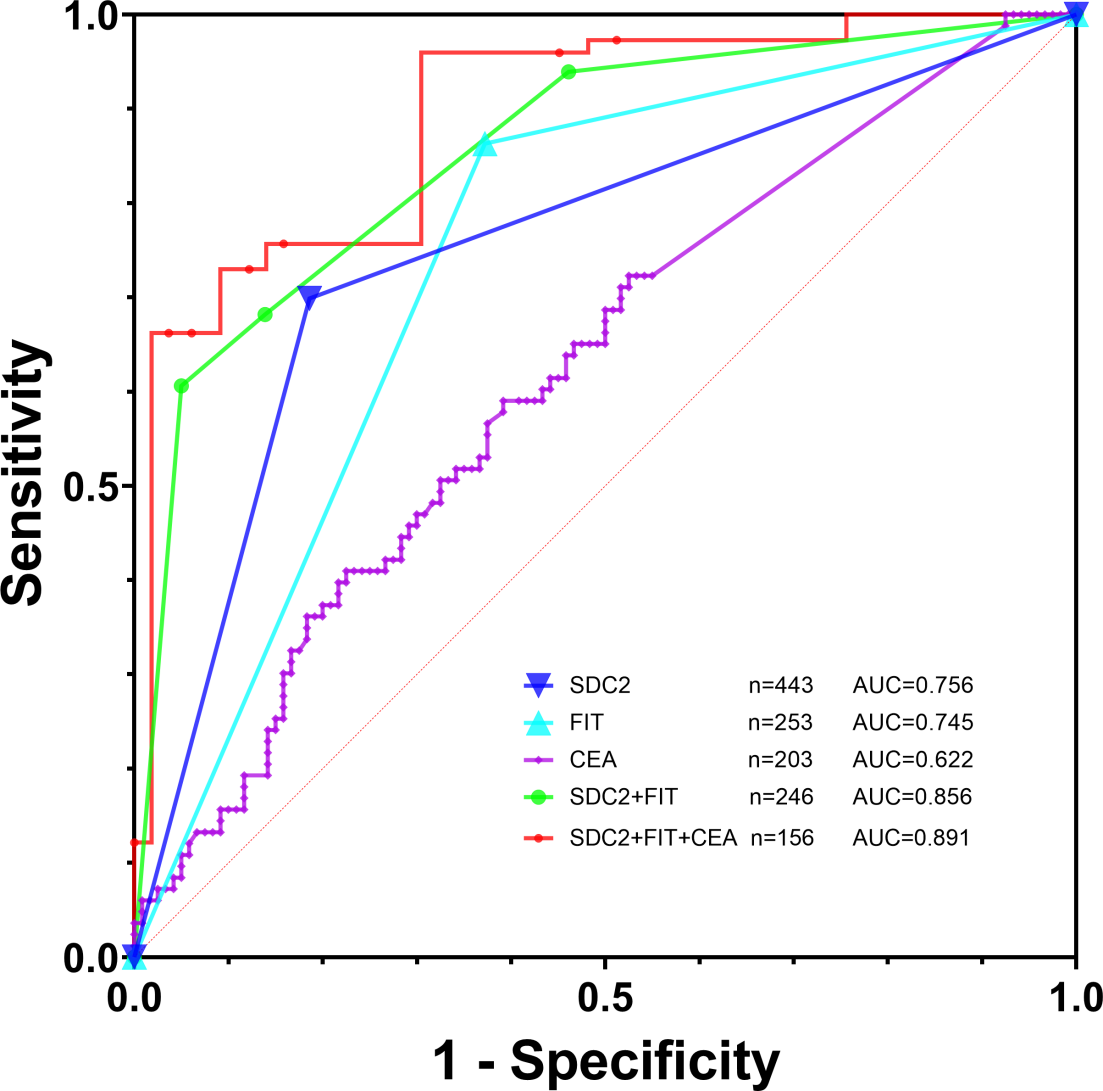
Efficacy of fecal SDC2, FIT, and tumor markers for early-stage CRC and APL diagnosis.

**Figure 5:**
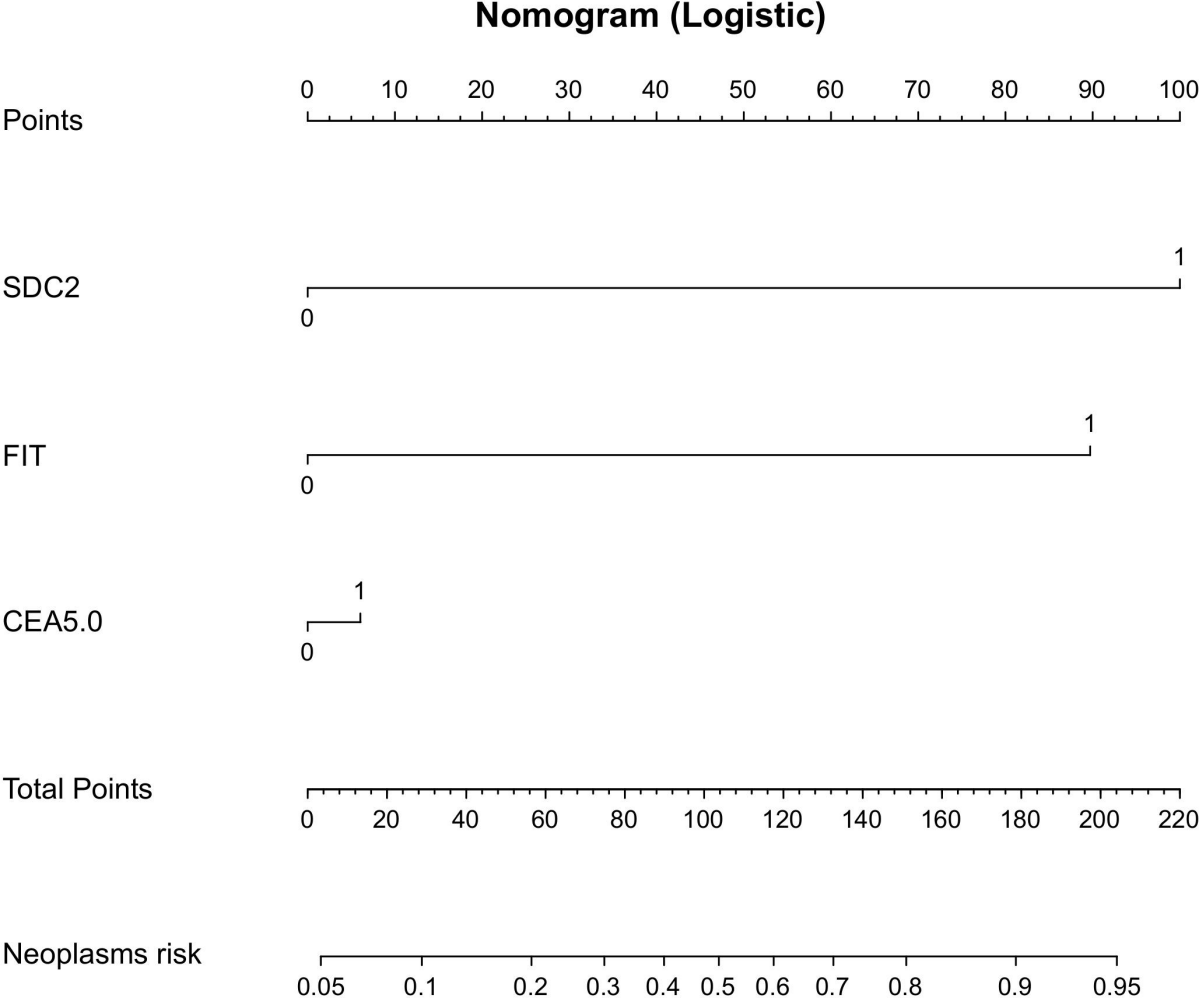
Nomogram-based prediction model for the risk of developing early-stage CRC and APL using logistic regression.

2. We then assessed the effectiveness of different tests in detecting early-stage CRC plus APL, with a control group consisting of non-AA and general polyps, benign lesions, and healthy controls. Analysis of SDC2’s efficacy in diagnostic early-stage CRC plus APL showed a sensitivity, specificity, and AUC values of 69.9%, 81.4%, and 0.756, respectively (**Table 4**; **Figure 4**). Detection efficacy for FIT in early-stage CRC plus APL revealed a sensitivity of 86.3%, a specificity of 62.8%, and the AUC of 0.745, indicating that the sensitivity of SDC2 was lower than that of FIT, but with higher specificity. Among the tumor markers, CEA showed a significant difference in the detection of early colorectal neoplasms (P = 0.0012, **Table 5**). The diagnostic efficacy evaluation of SDC2 combined with FIT showed a sensitivity of 60.6%, specificity of 95.0%, and AUC of 0.856. When combined with conventional FIT and serum CEA, the corresponding values were 66.2%, 96.3%, and 0.891, respectively. The coefficients for the independent variables, SDC2 and FIT, are 2.93287 and 2.6312, respectively. The coefficient for the CEA dependent variable is 0.17702. Finally, the model’s coefficient for the constant term is -2.7346. We have also created nomographs based on logistic regression, which are visually presented for the reference of clinicians (**Figure 5**).

**Table 4:** Efficacy of fecal SDC2, FIT, and serum CEA for early-stage CRC and APL diagnosis.

	No.	Sensitivity (%)	Specificity (%)	AUC (95%CI)	P value
SDC2	443	69.9%	81.4%	0.756 (0.714–0.796)	< 0.0001
FIT	253	86.3%	62.8%	0.745 (0.687–0.798)	< 0.0001
CEA	203	59.0%	60.8%	0.622 (0.551–0.689)	0.0018
SDC2 + FIT^1^	246	60.6%	95.0%	0.856 (0.806–0.897)	< 0.0001
SDC2 + FIT + CEA^1^	156	66.2%	96.3%	0.891 (0.832–0.936)	< 0.0001
SDC2 + FIT^2^	246	93.9%	53.9%	0.739 (0.680–0.793)	< 0.0001
SDC2 + FIT + CEA^2^	156	97.3%	48.8%	0.730 (0.654–0.798)	< 0.0001

^1^Use logistic regression to build prediction curves and ROC curve analysis to calculate the area under the curve. ^2^Result are considered positive if any one of them has a positive result.

**Table 5:** Efficacy of serum Tumor markers for early-stage CRC and APL diagnosis.

	No.	Sensitivity (%)	Specificity (%)	AUC (95%CI)	P value
CEA	193	58.0%	62.5%	0.628 (0.556–0.697)	0.0012
CA125	193	32.1%	76.8%	0.540 (0.467–0.612)	> 0.05
CA19-9	193	42.0%	67.0%	0.524(0.451–0.596)	> 0.05
CA15-3	193	55.6%	60.7%	0.575 (0.502–0.645)	> 0.05
AFP	193	95.1%	14.3%	0.516 (0.444–0.589)	> 0.05

3. Result was considered positive if any one of them has a positive result, our study found that the joint detection of SDC2 and FIT can detect more cases of colorectal neoplasms, with a combined sensitivity of 95.3%. In cases of early-stage CRC and APL, the sensitivity was 93.9%. However, the joint detection of SDC2 and FIT may result in lower specificity, leading to a higher rate of false positives (**Tables 3**, **4**). Therefore, the diagnostic efficacy of this method may be lower than that of SDC2 alone. When combined with CEA, the sensitivity of the detection method can reach its maximum, detecting up to 97.5% of colorectal neoplasms. Specifically, this method can detect 97.3% of early-stage CRC and APL cases.

## 4 Discussion

CRC is among the most common malignant tumors worldwide. Early screening can help to prevent and diagnose CRC, improve patient prognosis, reduce mortality, and lower the economic burden of disease on individuals (23, 24). The main detection method used is FOBT, followed by colonoscopy. FOBT is a cheap and convenient method for detecting gastrointestinal bleeding and is the most widely used screening method (25, 26). In this study, we utilized FOBTs for FIT, which could detect both hemoglobin (Hb) and transferrin (TF) without cross-reaction. Previous studies have mainly focused on Hb testing alone, while some studies suggest that TF testing can be an effective supplement for CRC and APL diagnosis. We routinely perform both types of FOBTs to effectively screen the target population (27, 28).

SDC2 methylation in stool DNA is an effective method for early detection of CRC (14, 29, 30); however, debates regarding the clinical application of fecal SDC2 are ongoing, and it has rarely been compared or combined with FIT to validate its screening efficacy. Given the effectiveness of individual target, the main use of multi-target stool DNA (4, 12, 31) or blood methylation tests (19, 32) for combined screening of colorectal neoplasms is currently in place, but their efficacy needs to be validated in large samples.

A previous multicenter study reported good diagnostic agreement between fecal SDC2 and colonoscopy, with a Kappa value of 0.84 (11). In contrast, our findings showed that the results of stool SDC2 were moderately consistent with colonoscopy (Kappa = 0.54; P < 0.001). This could be due to a higher number of false positives in the non-CRC and non-APL group in our study (25/180), potential selection bias due to the relatively small sample size, or a high specificity due to the inclusion of a majority of members of the healthy population in our prospective multicenter study. Meanwhile, our results showed that fecal SDC2 and FIT have different advantages and disadvantages for CRC detection. Further, the positive detection rate of SDC2 in colorectal adenocarcinoma was 77.8% (70/90), while that of FIT in adenocarcinoma was 97.8% (88/90); and the positive detection rates of fecal SDC2 and FIT in poorly differentiated CRC were both low, at 66.7% and 88.9%, respectively, suggesting that for poorly differentiated CRC it is necessary to combine these methods with other approaches. Fecal SDC2 and FIT were able to detect more proximal tumors. The positivity rate for FIT was higher than that for SDC2 in both proximal and distal areas, with a positive detection rate of over 90% in both regions. The detection rate of fecal SDC2 decreased with increasing depth of tumor infiltration, from 89.3% at T0-2 to 75.9% at T3-4. Conversely, the detection rate of FIT was higher at T3-4 than at T0-2. The positive detection rates of fecal SDC2 and FIT were higher in patients with lymph node and distant metastasis than in those without metastasis. This suggests that positive fecal SDC2 and FIT results may be able to predict a poor prognosis for patients, although more evidence is needed to verify this hypothesis. Based on the relevant guidelines (5), FIT has high sensitivity for CRC diagnosis, but limited sensitivity for APL, which is also consistent with our results.

Our data show that the combination of fecal SDC2 and FIT improved the positive detection rates for early-stage and advanced CRC and APL to 98.0% (48/49), 98.4% (60/61), and 82.4% (14/17), respectively, suggesting that the two methods are complementary (**Table 1**). It is evident that the combined SDC2 and FIT test exhibited a higher detection rate for CRC and APL. Only 6 out of 128 colorectal neoplasms were not detected, indicating a remarkably high sensitivity. Given the widespread use of tumor markers in clinical practice, our data demonstrate a significant difference in CEA levels between early colorectal neoplasms and controls (**Table 5**). In relation to the combined SDC2, FIT, and CEA test showed a detection failure in only 3 cases out of a total of 120 colorectal neoplasms, thereby further enhancing the sensitivity. This result suggests that patients with colorectal neoplasms who test negative for CEA screening may benefit from detecting SDC2 and FIT, which may increase the positive detection rate. The data presented in **Tables 1**, **3** exhibit consistency and mutual verification. The corresponding data strongly indicate that the combination of the three tests successfully detected a maximum number of patients. The cost-effectiveness of diagnostic tests is a crucial factor to consider in clinical practice. Based on our calculations, the cost of painless colorectal microscopy and the cost of SDC2 methylation, FIT, and CEA are relatively similar (all around 1000 RMB). However, other factors such as test simplicity, patient acceptability, operability, and large-scale scalability should also be considered. Therefore, clinical practitioners should develop different screening strategies tailored to the specific needs of their patients.

According to expert consensus (33), FIT is not currently recommended as a screening modality for APL. Biomarker combinations have been shown to have better screening efficacy than individual markers, and the combined use of fecal and blood markers can improve the sensitivity for detecting colorectal neoplasms. Additionally, alterations in gut microbiology may also influence the development and progression of CRC (34).

Our study investigated the diagnostic effectiveness of combining SDC2, FIT, and CEA using two methods. First, we evaluated each indicator’s clinical significance, alone and in combination, using logistic regression and ROC curve analysis. We found that the combined detection curve had the highest AUC at 0.891 in early-stage CRC and APL, while the AUC for a single CEA indicator was only 0.628. Second, we used a simpler approach that considered any of the indicators as positive, but this led to more false positives, mainly due to FIT’s lower specificity. As a result, combining detection did not improve diagnostic efficacy and even performed worse than using SDC2 alone. Sensitivity refers to the probability of a classifier accurately predicting positive values among all positive samples. The AUC takes into account both true positive and false positive rates, making it a more comprehensive evaluation index for assessing a model’s predictive performance. However, clinicians should choose suitable methods based on their purpose and practicality when making decisions. Our test results can serve as a reference, but additional examinations such as imaging or pathology must be combined to make a comprehensive diagnosis.

## 5 Conclusions

Fecal SDC2 is useful for early screening of CRC and APL. Combining SDC2 with FIT (Hb and TF) can improve the positive detection rates of early and advanced CRC and APL. Additionally, combining fecal SDC2 and FIT with serum CEA has shown high detection efficacy. Using a combination of these methods could be a new approach for early screening of CRC and APL, but its effectiveness requires further validation in large sample populations.

## Data availability statement

The raw data of this study are stored in the Research Data Deposit (RDD) of Sun Yat-sen University, which can be requested from the author, TZ with reasonable request.

## Ethics statement

This study was approved by the Ethics Committee of The Sixth Affiliated Hospital of Sun Yat-sen University, Ethics No. 2022ZSLYEC-508. Written informed consent has been obtained from the individual(s) or their legal guardian/next of kin or from the patients/participants in this study.

## Author contributions

TZ: Research design, research implementation, data collection, data analysis, article writing. ZH: Research implementation, data collection. XY: Research design, research guidance; LZ: research guidance, data collection. TL: Research design, research guidance, article review and revision. BT, SX, JH: Research design, research implementation, research guidance, data analysis and interpretation, article review and revision. 
